# Two genes, one switch: a bidirectional promoter strategy for inducible plant immunity

**DOI:** 10.1093/plphys/kiag243

**Published:** 2026-04-24

**Authors:** Benjamin J M Tremblay

**Affiliations:** Assistant Features Editor, Plant Physiology, American Society of Plant Biologists, Rockville, MD, United States; The Sainsbury Laboratory, University of East Anglia, Norwich Research Park, Norwich NR4 7UH, United Kingdom

Plant diseases remain a major threat to food security, reducing crop yield worldwide. A central challenge in crop protection is that effective immunity often comes at a cost. Plants must continuously balance growth with defense, and this balance complicates molecular breeding strategies aimed at improving disease resistance. When immune pathways are constitutively activated, plants are often better protected against pathogens; however, the price for this protection is reduced growth or fitness. This defense-growth tradeoff has therefore become a major obstacle to engineering disease resistance in crops (Han et al. 2024).

Plants rely on a multilayered immune system to detect and respond to infection (Jones and Dangl 2006). At the cell surface, pattern-recognition receptors sense conserved pathogen-associated molecular patterns and activate pattern-triggered immunity (PTI), which includes outputs such as reactive oxygen species production and defense gene expression. Inside the cell, nucleotide-binding leucine-rich repeat receptors (NLRs) detect pathogen effectors and activate effector-triggered immunity, a stronger response that can include localized programmed cell death. Although these pathways are highly effective, their activation must be tightly controlled because untimely or persistent defense signaling can impair normal development (Karasov et al. 2017).

One promising solution is to place immune genes under the control of promoters that are activated only when defense is needed. Such immune-inducible promoters could provide “on-demand” resistance, reducing the fitness costs associated with constitutive expression. This idea joins a growing set of strategies aimed at minimizing the defense penalty, including the exploitation of natural variation, the engineering of immune receptors, the editing of susceptibility genes, and translational control approaches such as immune-inactivated upstream open reading frames. However, beyond inducible gene expression, durable resistance may require the coordinated expression of multiple defense genes, but stacking genes under repeated use of the same promoter can increase the risk of transgene silencing.

Bidirectional promoters could help solve this problem. Many plant promoters are inherently bidirectional, transcribing both a protein coding transcript in one direction and a noncoding in the other (Tremblay et al. 2024), but relatively few well-characterized examples are known in which 1 promoter coordinates the expression of 2 protein-coding genes. Previous studies have shown that bidirectional promoters can be useful tools in plant biotechnology, suggesting a possible route toward synchronized expression of paired defense genes (Bai et al. 2019; In et al. 2020). The key question, however, is whether naturally occurring immune-inducible bidirectional promoters exist that are strong, tightly regulated, and suitable for engineering disease resistance.

Recently in *Plant Physiology*, Lin et al. (2026) addressed this question by searching for promoters that could drive inducible expression of multiple resistance genes simultaneously. Using transcriptome analysis of Arabidopsis seedlings treated with PTI elicitors flg22 and chitin, the authors identified 7 bidirectional promoters associated with defense-responsive gene pairs encoding a range of functions. To test promoter activity, the authors built a dual-reporter system in which each orientation of the bidirectional promoter drove a distinct fluorescent protein (Figure 1). These constructs were transiently expressed in Arabidopsis protoplasts, allowing quantitative measurement of inducibility and promoter symmetry under immune-eliciting conditions. Among these, one promoter stood out because it showed strong activation in response to flg22 and chitin while maintaining low basal expression in their absence. The inducible behavior was also validated in stably transformed plants, indicating that its performance was not limited to transient assays. The strongest promoter, a 313-bp sequence found between the immune signaling receptor-like kinase *SUPPRESSOR OF ZED1-D1* (*SZE1*; *AT5G25440*) and a mitochondrial cytochrome bd ubiquinol oxidase (*QCR7-2*; *AT5G25450*) termed BiPro1, was not only activated by flg22 and chitin but also by infection with bacterial pathogens, *Pseudomonas syringae* pv. *tomato* DC3000, *Ralstonia solanacearum*, and the necrotrophic fungus *Botrytis cinerea*. These tests confirmed that BiPro1 responds robustly to a broad range of pathogens.

**Figure 1:**
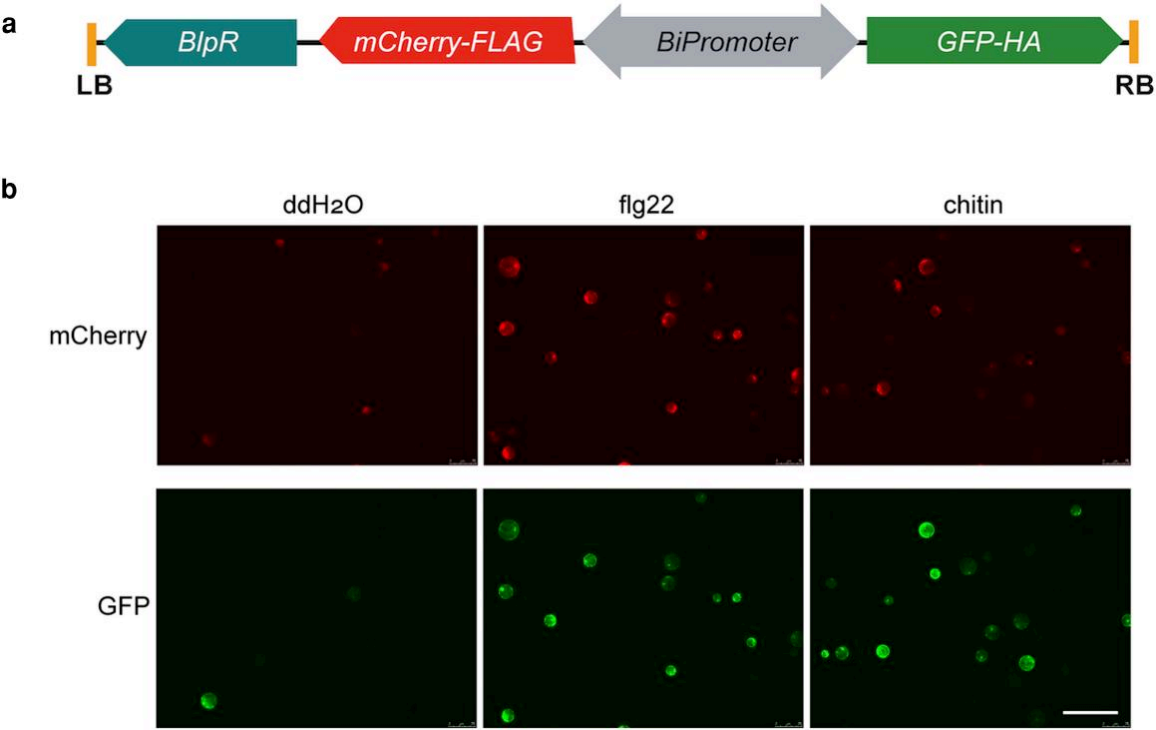
Validation of immune-inducible promoter bidirectionality. a) A dual reporter system in which a single promoter sequence drives the expression of fluorescent proteins *mCherry-FLAG* and *GFP-HA*. b) Using this reporter system, the BiPro1 bidirectional promoter displayed low basal expression when transiently expressed in Arabidopsis protoplasts but was strongly induced by immune-elicitors flg22 and chitin. Adapted from Figure 2 of Lin et al. (2026).

The authors next asked whether this promoter could be used to control a pair of immune receptors in their native genomic context. To do this, they used CRISPR/Cas9-mediated homologous recombination to replace the intergenic region between the head-to-head NLR genes *RRS1* and *RPS4* with the selected immune-inducible bidirectional promoter. This choice was motivated by the fact that *RRS1* and *RPS4* function together as a receptor pair and are naturally arranged in divergent orientation (Narusaka et al. 2009). While RRS1 and RPS4 are likely co-regulated via a shared short promoter region (Narusaka et al. 2013), they are only weakly induced during PTI (Lin et al. 2026). In the edited plants, expression of the 2 NLR genes remained low under non-induced conditions but increased upon elicitor treatment, demonstrating that the engineered promoter could coordinate their inducible expression in planta.

Crucially, this system appeared to mitigate the classic defense-growth tradeoff. Plants carrying the engineered promoter grew normally, suggesting that low basal expression was sufficient to avoid the developmental penalties often associated with constitutive NLR overexpression. At the same time, these plants displayed enhanced resistance to both bacterial and fungal pathogens, likely due to slightly higher expression of some defense genes upon attack compared with unedited plants. Together, these findings provide a compelling proof of concept that bidirectional immune-inducible promoters can be used to coordinate defense gene expression while limiting the costs to plant growth.

A key question raised by this work concerns mechanism: why does increased expression of NLRs that do not directly recognize the tested pathogens still improve disease resistance? The authors suggest a model based on recent evidence that several TIR-type NLRs are rapidly induced by elevated cytosolic Ca^2+^ during early PTI and generate small signaling molecules via their NADase activity that enhance EDS1-dependent defense gene expression activation (Wan et al. 2019; Tian et al. 2021; Maruta et al. 2026). By mimicking this endogenous pattern, BiPro1-mediated induction of the TIR-type NLRs *RRS1* and *RPS4* by flg22 or chitin may further boost the immune system's ability to respond more vigorously to infection.

Another open question relates to promoter biology itself. Although bidirectional transcription is widespread in eukaryotes, the sequence features and chromatin environments that enable some promoters to drive robust expression in both directions remain poorly defined. Understanding how immune activation modulates these promoters could advance synthetic biology by informing the design of regulatory elements with tuned strength, symmetry, or elicitor specificity.

Finally, the study suggests broader opportunities for translation. Many plant NLR genes occur in head-to-head arrangements, raising the possibility that similar promoter replacement strategies could be deployed for receptor pairs in other crop species, bypassing endogenous tissue-specific expression patterns or suppression of NLR expression by pathogen effectors (Fick et al. 2022). Whether this approach will generalize across diverse genomes and infection scenarios remains to be tested, but the concept is highly appealing. Rather than constitutively expressing large gene stacks, breeders and engineers may be able to insert compact regulatory sequences that improve activation of endogenous defenses upon attack.

In summary, Lin et al. (2026) show that immune-inducible bidirectional promoters can provide coordinated, low-cost activation of disease resistance genes. By combining transcriptome-guided promoter discovery with precise genome editing, they offer a practical strategy to improve resistance while avoiding the growth penalties that limit immune engineering. More broadly, the work positions bidirectional promoters as promising tools for building the next generation of disease-resistant crops.

## Recent related articles in *Plant Physiology*:

- Li et al. (2024) edited an immune receptor to mimic a constant primed state in crops to improve pathogen defense without a growth penalty.
- Tian et al. (2025) modified the upstream open reading frame of a defense gene to boost its protein levels and increase pathogen resistance in rice.

## Data Availability

None required.
